# Impact of COVID-19 on children

**DOI:** 10.1186/s43045-022-00256-3

**Published:** 2022-11-16

**Authors:** Ulfat Amin Amin, Asmat Parveen Parveen

**Affiliations:** SMMCNMT, Islamic University of Science & Technology Srinagar, Jammu and Kashmir Awantipora, India

**Keywords:** COVID-19, Vaccination, Impacts, Prevention

## Abstract

**Background:**

The COVID-19 global pandemic has spread throughout the world, posing an extremely dangerous health risk for almost everyone. While dealing with such a large-scale viral disease, the healthcare infrastructure is under strain. Young adults who were thought to have been clinically affected fared better than their older counterparts. This pandemic has affected millions of children, especially those from low-economic backgrounds, who are otherwise highly susceptible and underprivileged. Children of frontline workers and single parents face particular challenges. Children from disadvantaged backgrounds are more vulnerable to infection and may experience long-lasting negative effects of the pandemic, such as child labor, child trafficking, child marriage, sexual exploitation, and even death. To lessen the psychological negative effects of COVID-19 on children and adolescents, parents, physicians, psychologists, social workers, and hospital administrators, government and non-governmental organizations have essential responsibilities to play. Priority one is to ensure that all children from all socioeconomic strata have access to the necessities of life, including social security, health care, and education. Moreover, some positive changes may result from the global crisis. This research paper discusses the potential consequences of this pandemic.

**Summary:**

Some of the hypotheses being investigated while looking at the low case fatality rate among pediatric age groups include the peak of immunity and differences in immune system response. The vulnerability of the comorbid pediatric age group, on the other hand, is comparable to that of their older counterparts. During the severe acute respiratory syndrome and Middle Eastern respiratory syndrome outbreaks, similar results were observed. The inoculation of a mother during pregnancy was found to be effective in protecting her progeny.

**Results:**

This is a review article, thus not applicable.

**Conclusions:**

Children are quite discouraged when school is abruptly stopped, when planned outings are canceled, when they are confined to the house, and when they are afraid of the unknown with regard to the continuing epidemic. Even though almost all studies suggest that COVID-19 has a relatively mild clinical manifestation in children, one must be cautious due to the novel coronavirus’s rapid mutation rate. More research is needed to determine the relationship between COVID-19 and pediatric age groups.

## Background

COVID-19 is an illness that is caused by a novel coronavirus known as SARS-COV-2 that has been causing chaos around the world. Controlling the spread is more difficult due to the highly infectious nature of the disease and its ability to cause clinical complications in patients. It has grappled the entire world since its inception in Wuhan, Hubei province of China, reaching every nook and corner and contaminating people from all walks of life. Every person has been impacted in some way, most often negatively. COVID-19-related complications have resulted in 505,035,185 infections and 6,210,719 case fatalities as of 21 April 2022 [1]. COVID-19 in the pediatric age group is a hot topic of research because it affects the bulk of the population in many countries. Previous SARS and MERS outbreaks have taught us valuable lessons and shed light on a previously unknown aspect [2]. Due to their immunosuppressive state, pregnant women are considered a vulnerable segment of the population. This can have a significant impact on the newborn baby in a variety of ways. Inoculation of children and young adults has only recently begun in a few countries, so it is crucial to understand the link between COVID-19 and its clinical manifestation. COVID-19’s long-term implications and etiopathogenesis are causing stress and anxiety in the teenage population [3]. Preventative measures are an effective way to stop the spread of a virus. This article takes a comprehensive look at all of these factors.

## Methods

**Fig. 1:**
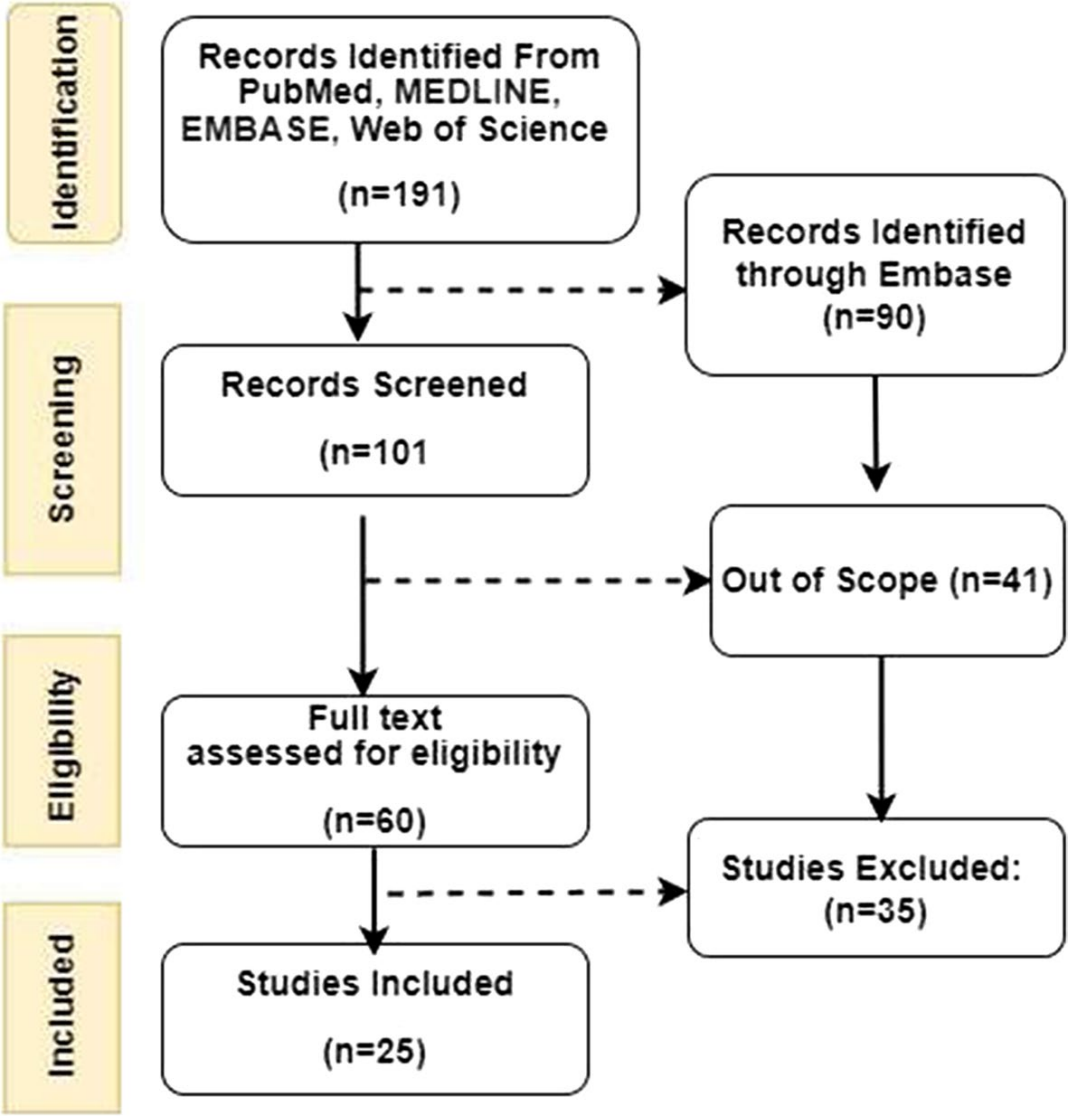
Identification of studies via database and registries

Following the standards of PRISMA review of the literature was taken from MEDLINE, EMBASE, Web of Science, and PubMed databases. According to the Medical Subject Headings (MeSH) keywords like, “impact of COVID on children,” “psychological impacts,” “negative impacts,” “quarantine,” “SARS-CoV-2,” “COVID-19,” “nursing care,” “nursing management,” “clinical management,” and “infection control and prevention in COVID-19,” were utilized as key phrases. From the beginning, studies in English were included. Gray literature was also reviewed to gain additional knowledge regarding the epidemiology and management of this unique illness (Fig. 1).

## Clinical manifestation

COVID-19 is a highly contagious viral pandemic that is rapidly spreading. It is caused by SARS-COV-2, a novel coronavirus that is the newest member of the coronaviridae family. Severe acute respiratory syndrome (SARS) and Middle Eastern respiratory syndrome (MERS), both caused by members of the coronaviridae family, have caused less harm than SARS-COV-2. The novel coronavirus has unrivaled infectiousness and the ability to cause comorbidities in the infected person’s body. The high death toll and infection cases caused by COVID-19 complications can be attributed to this fact. The virus is primarily transmitted through the air and can remain suspended in the atmosphere for long periods of time. It infects humans primarily through physiologic entrances such as the oral cavity, nose, and eyes, and attacks the mucous membranes of the upper respiratory tract [4, 5]. By taking swabs from the oral and nasal passages, the reverse transcriptase polymerase chain reaction (RT-PCR) test is used to determine whether the patient is positive or not. The symptoms appear 4 to 12 days after the infection and differ from person to person. Fever, cough, and cold are common symptoms, but they can also include diarrhea, dyspnea, dysosmia, and other symptoms. Infants were thought to be particularly vulnerable to infection because their immune systems are still developing and in the nascent stage, and they are exposed to the external environment. In fact, in some countries, all pregnant mothers were advised to postpone their pregnancy because the health care system was not prepared to tackle the huge surge if the virus spreads to both mother and child. But, in reality, the facts gathered contradicted this theory. The mothers-to-be were tested at various stages of their pregnancy. Pregnant mothers are particularly vulnerable to COVID-19 infection because they are already immunocompromised [6, 7]. A study was carried out to discover the truth about perinatal and neonatal outcomes. To debunk the link between COVID-19 and pregnant mothers and their offspring, researchers looked at 388 pregnancies. The information was collected from 22 various countries and more than 70 COVID-19 care centers [8, 9]. The majority of pregnant women were diagnosed in the third trimester, with only 8% and 22% of pregnant women being diagnosed in the first and second trimesters, respectively. More than 24% of expectant mothers were asymptomatic, while the rest had symptoms such as cough, cold, and fever, as well as shortness of breath on occasion. Although shortness of breath is commonly observed in pregnant women, it is unclear whether this is due to COVID-19 or pregnancy. 11.1% of infected pregnant women were admitted to intensive care unit (ICU). In 17 cases, there was a negative fetal outcome, while in the other 248 cases, there was no negative fetal outcome [10]. Although it was unclear whether the negative fetal outcome was caused by COVID-19 contractions during pregnancy or something else. Perinatal death occurred at a rate of 4%, which was attributed to prematurity. Although no concrete evidence of COVID-19 infection transmission from mother to child has been discovered. Vertical transmission refers to the transmission of an infection from a mother to her offspring via any means, such as the placenta. Although the vertical transmission phenomenon has been widely discussed, no concrete evidence has been found to back it up. In fact, infants who tested positive for COVID-19 were found to be recovering on their own, with no significant negative consequences. Neonates and infants as compared to toddlers and school-age children are more likely to develop serious clinical consequences as a result of COVID-19 infection because their respiratory tracts are still developing and highly susceptible, unable to tolerate the immune response [11]. The infection can be contracted in a variety of ways by newborn babies. To begin, the COVID-19 infection can be transmitted to a newborn baby through physical exposure to an infected mother. Even though neonates are kept away from COVID-19-infected mothers, certain conditions such as breastfeeding can transmit the disease to the baby.

The unhygienic conditions in the hospital led to the spread of disease from hospital staff to newborns again. Even though babies tested negative in most cases, some suffered complications that could have been prevented by strictly following operating procedures. A similar trend was found in another compilation of cohort studies worldwide. Patients from the USA, China, and Italy, who tested positive for COVID-19, were evaluated. Nearly 50% of patients were reported to have been in contact with another infected person. Cough and cold were the most prevalent symptoms. Among the patients studied, there was no vertical transmission reported [12].

### Impact of COVID-19 among the pediatric age group

#### Positive impacts

##### ***Increased awareness***

**Fig. 2:**
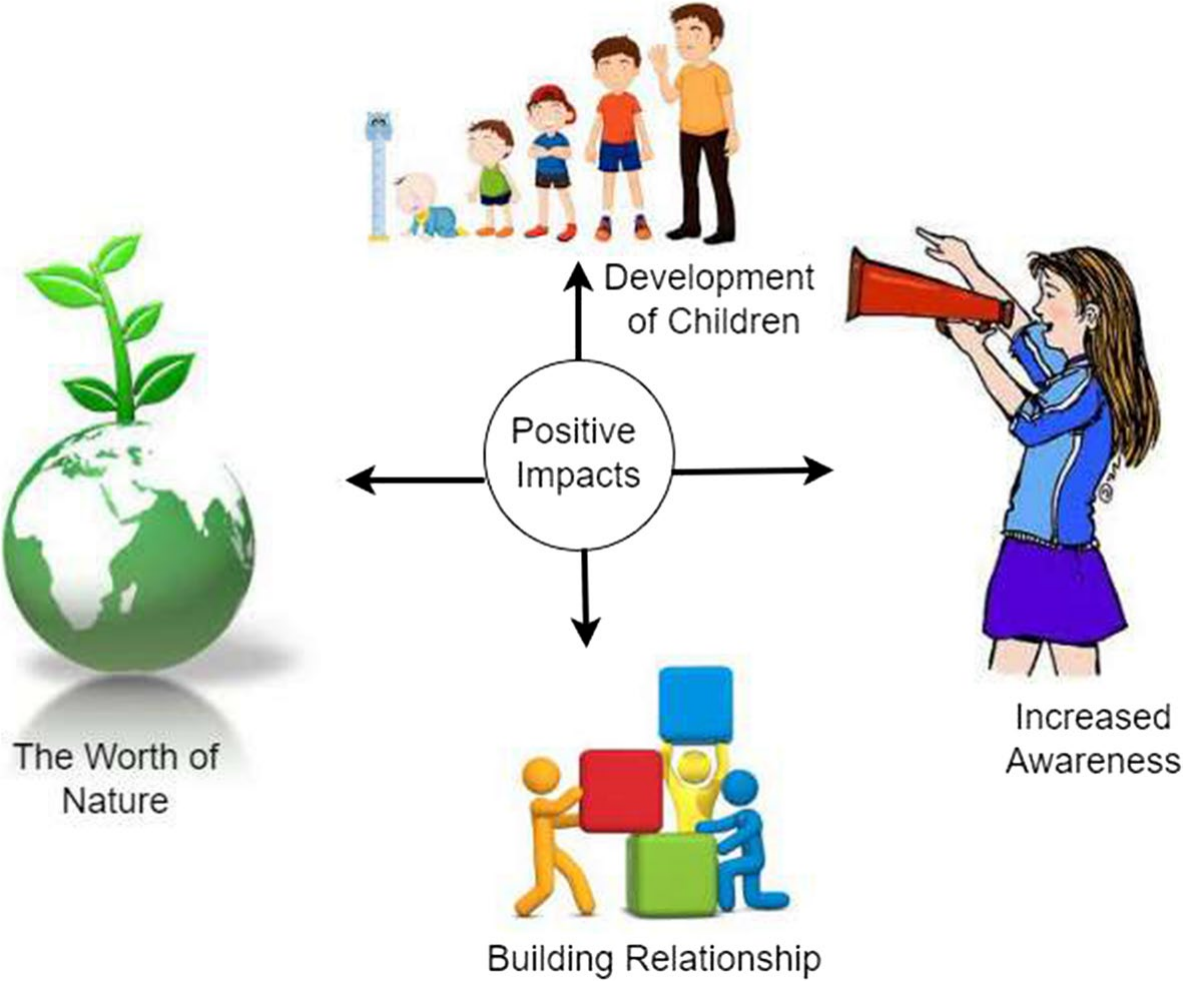
Positive impacts of COVID on children, the image was drawn by the author

UNICEF, the World Health Organization, and health authorities have urged parents to talk to their children about the pandemic in precision. UNICEF, for example, has created eight top tips for assisting and comforting children during the pandemic. Parents should be open and honest with their children, as well as reassure them and explain what practical steps they can serve to keep themselves and others safe [13, 14] (Fig. 2).

##### ***Building relationship***

Investing time with family members may help several children form closer bonds with them, and being aware of the pandemic's impact may teach children more morality and compassion as they recognize the humanity [15].

##### ***Development of children***

School closures are expected to harm children’s education. UNESCO has strongly supported distance education solutions, including the use of digital teaching aids. Children who have direct exposure to that will be capable of learning instructional strategies that would help them later on in life. During this, they could also participate in a variety of physical, learning, and creative activities that will help them develop their skills [11, 16].

##### ***The worth of nature***

Because there is less traffic, there is less pollution and noise. Anecdotal evidence suggests that this has resulted in changes in our natural surroundings and given children more possibilities to see and acknowledge biodiversity.

#### Negative impacts

##### ***Impacts on education***

**Fig. 3:**
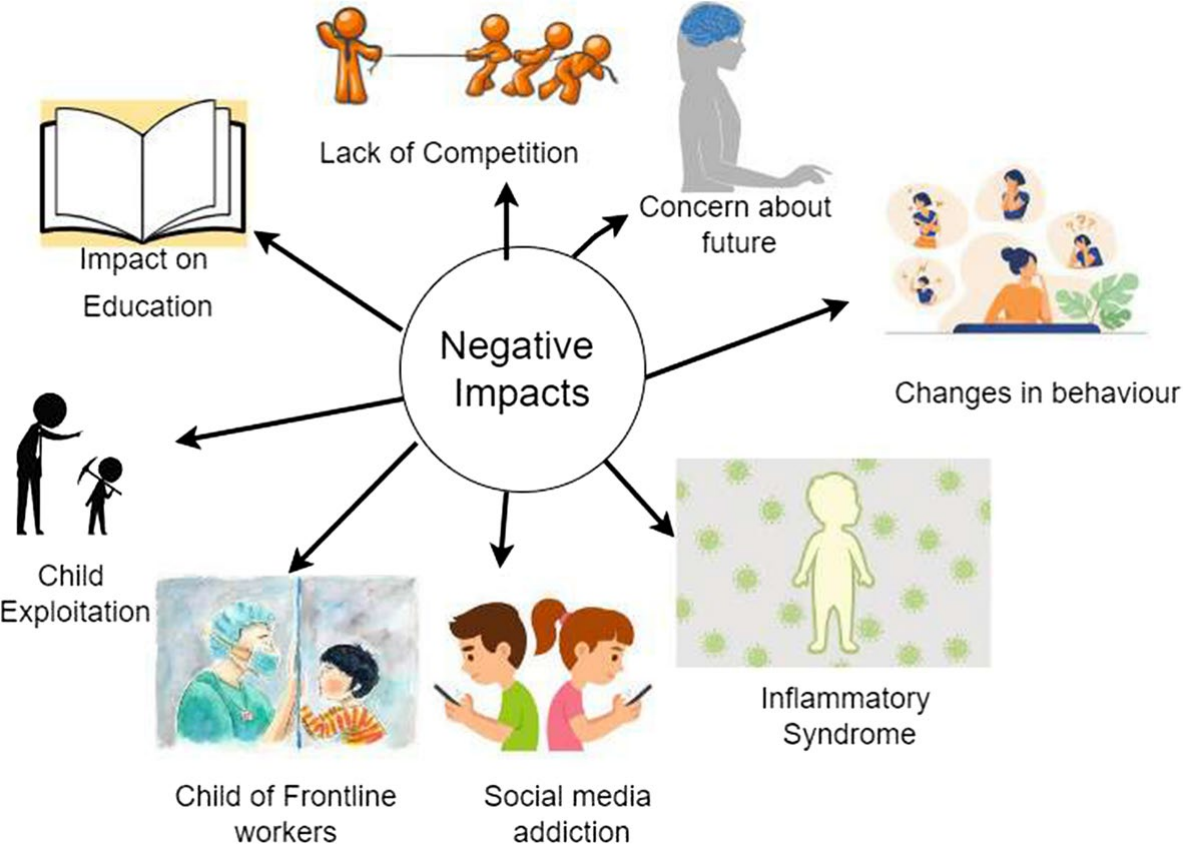
Negative impacts of COVID on children, the image was drawn by the author

Closing schools and libraries, which are the only places where some children can get access to the Internet, will have a variety of effects on children’s future. Non-school factors, according to Lancker and Parolin, are a major source of educational inequity. They believe that, as a result of school closures, focusing on digital education will exacerbate the knowledge gaps among children from low and high economic classes. In fact, they have labeled the combination of closing schools and poverty as a looming national crisis [17]. We believe that the most severe consequences will be felt in the lowest income groups and rural areas with no or slow Internet access. As a result of the economic downturn, families may face difficulty affording broadband services (Fig. 3).

##### ***Lack of competition***

Schools provide social and competitive activities that many children enjoy, and interacting with children from various backgrounds helps them adapt to new situations and form social bonds. During the pandemic, Viner et al. conducted a rapid systematic review of 16 papers and concluded that school closures and social isolation could impede children’s psychological and personal development.

##### ***Concerns about the future***

Exams have now been deferred or postponed as a result of the pandemic, and some students may be concerned about their future. These issues may also cause them to miss out on further academic achievement or the start of their working life.

##### ***Changes in behavior***

Detachment, physical separation, and solitude are all difficult situations for anyone. Children miss communication with their peers, and we fear that being separated from them for an extended period will result in drastic behavioral changes [18].

##### ***Internet and social media addiction***

Many children were actively encouraged to go online to continue their education due to school closures. They may also be subjected to offensive content and cyberbullying. According to a study conducted before the pandemic, social media exposes children to a higher risk of cyberbullying, which can lead to stress, anxiety, low self-esteem, and even suicide attempts [19]. Inappropriate content and conversations, such as sexual images and pornography, are among the dangers of being online. For children, social networking could be a major source of addiction to a variety of hazardous substances. In their study, Primack et al. found evidence that alcohol promotions influenced a proportion of students to drink [20]. According to Ray and Ramjat’s research, there is a strong link between adolescent smoking and familiarity with advertising messages [21].

##### ***Child exploitation***

At the time of the COVID-19 pandemic and shutdown, reports of child maltreatment, abandonment, extortion, and domestic abuse are at an all-time high [22]. The COVID-19 pandemic, according to UNESCO, had a catastrophic influence on children in disadvantaged communities, particularly females [23]. Misuse, mental violence, and physical reprimand of children by caregivers at a young age leave enduring scars in the form of delayed intellect and personality development, greater incidence of psychological and neurological illnesses, various addictions, and suicidal tendencies [24, 25].

##### ***Children of frontline workers***

Although periods of shutdown provide an excellent opportunity for parents to interact with their children, parents who provide continuous health and defense services are time-poor, restless, and under-resourced, with little time for their families and children. The fear and guilt of infecting their children with a terrible illness is wearing them down. Breastfeeding, a basic requirement for raising a kid, is threatened if the woman is a frontline health professional [26].

##### ***Inflammatory syndrome***

It was reported that several children responded differently to the novel coronavirus infection, they got some autoimmune problems, making it difficult for them to get treated. Another syndrome that emerged in children after COVID-19 was systemic inflammatory disorder, in which high levels of inflammatory response have been seen in various vital body organs such as the liver, cardiovascular systems, heart, urinary system, gastrointestinal tract, nervous system, skin, and eyes, among others [27, 22]. Long-lasting pyrexia, severe stomach discomfort, diarrhea, red eyes, tachypnea, irritation and swelling on the lips and tongue, high degree of weariness, headaches, and other signs and symptoms can all be associated with MIS-C. A doctor should be consulted right away.

##### ***Impact of COVID-19 on specially abled children***

Children with disabilities are more susceptible because of their social environments as well as their underlying medical issues. In addition to having a greater chance of getting COVID-19, they are more likely to be poor and of a minority race (and as a result, suffer the negative effects of institutional and personally mediated racism more often than their non-disabled peers) [28, 29]. Additionally, they often have several overlapping identities that are linked to worsened inequities, making them and their families targets of stigma and discrimination [30, 31]. The social activities of and possibilities available to children with a range of impairments are often restricted [32]. Even when their children are covered by Medicaid, families with disabled children have greater rates of job loss and financially taxing medical expenses [33]. High rates of unmet requirements and poor access to high-quality healthcare are widespread problems with clear room for improvement [11]. The COVID-19 epidemic has made the already challenging circumstances faced by families with disabled children even more challenging.

#### Psychological impact

The worry of developing a sickness is on a whole different level, and it causes a lot of anxiety. Preliminary research of 320 youngsters in China indicated a high level of irritation, restlessness, distraction, and fear of a pandemic, all of which are negatively impacting mental health. Mental health is rarely mentioned in schools [34]. Stress can leave a youngster scarred for life, and it can do irreversible damage in these uncertain times. Long periods spent on cell phones while schools and colleges close due to non-pharmaceutical interventions, a void in social life, and maybe impaired study and career prospects all contribute to an increase in negative thinking [35]. This is exacerbated when a family member becomes afflicted, leaving the child helpless and hopeless. COVID-19’s clinical presentation not only bothers patients during therapy, but also bothers them after they have recovered [28, 36].

**Fig. 4:**
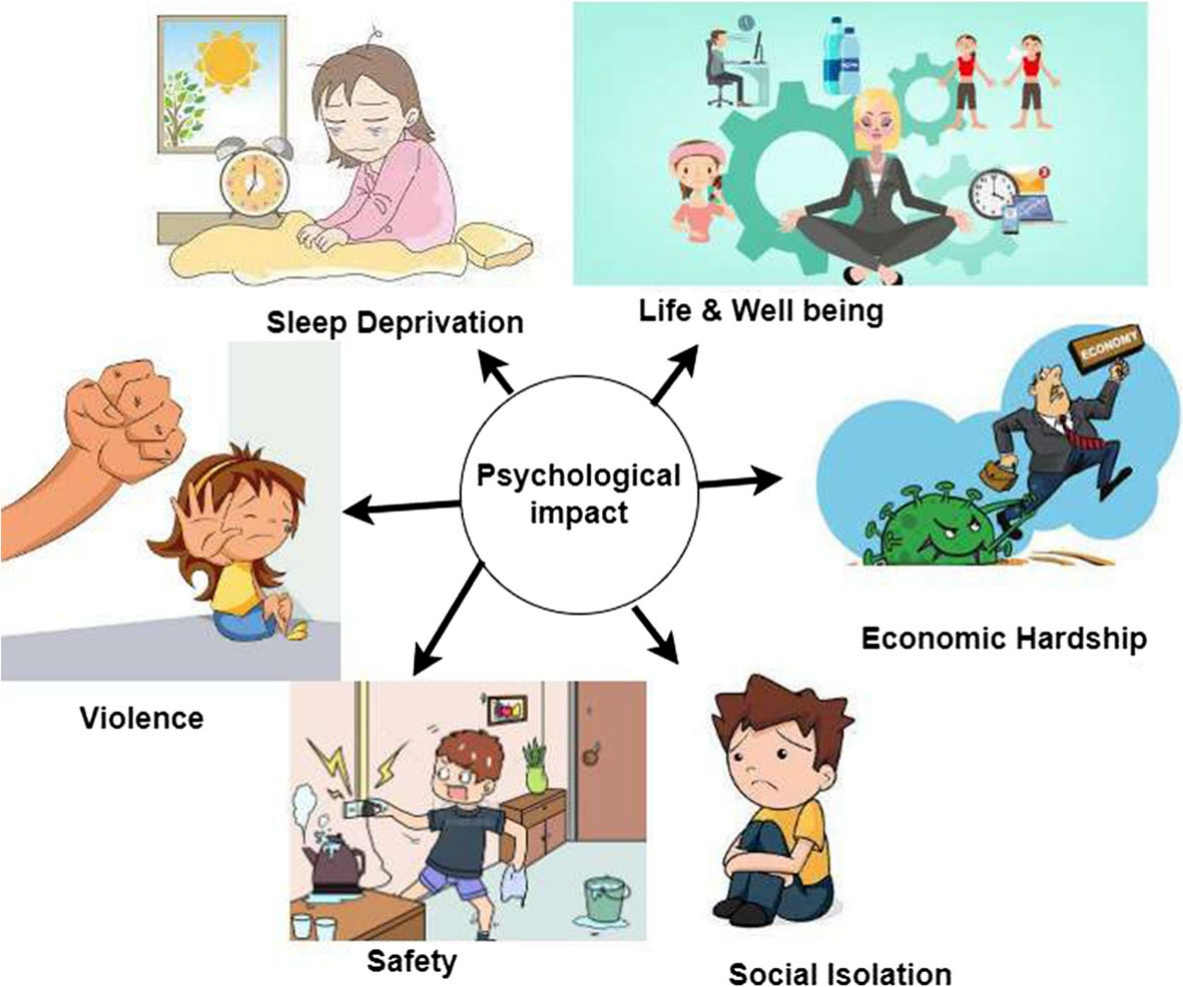
Psychological impacts of COVID on children, the image was drawn by the author

As the pandemic still continues to expand, dread of the outbreak, home confinement, and lifestyle changes are definitely causing a psychological impact on children (Fig. 4). Some of these are as follows:

##### ***Sleep disturbance***

The prevalence of sleep problems in children and adolescents during the COVID-19 pandemic is alarming [37]. The current COVID-19 outbreak seems to have been accompanied by a lot of sleep issues. Additionally, it was shown that sleep issues were linked to greater levels of psychological discomfort [38]. There were 55.5% of schoolchildren 6–12 years of age who reported having trouble sleeping during the COVID-19 pandemic [39].

##### ***Sliding into economic hardship***

At a household level, the decline in income threatens the welfare of millions of households with children throughout the globe. Inputting the estimates from the International Monetary Fund optimistic scenario into the International Food Policy Research Institute poverty model indicates an increase in dire poverty (PPP$1.90 a day) this year of 84 to 132 million people, approximately half of whom are children, compared to a pre-pandemic counterfactual scenario. Such revenue surges at the domestic level, although only temporary, can also have disastrous impacts on children, especially ones living in poor households with constricted assets [40–43].

##### ***Violence***

Many children and their families may have experienced significant changes as a result of the COVID-19 pandemic, not only because of the lockdown, restrictive measures, social isolation, shifting demographics, and the reduction of health care services [32], but also because of the abrupt and potentially long-lasting rise in childhood poverty and family uncertainty [44]. Through a sliding process of elements that might generate, induce, or amplify potential stresses, the pandemic symbolizes a worldwide issue that affects not just our health and economy but also our family well-being. Due to the COVID-19 crisis’ unprecedented situation, children and adolescents are at a high risk of being victimized [11, 15].

##### ***Life and wellbeing***

Children have so far experienced the direct effects of COVID-19 infection significantly less severely than other age groups. In contrast to COVID-19’s direct impacts, the pandemic’s wider consequences on children’s health are important [45, 31]. Reduced family income will require low-income households to make cuts to necessary food and health care expenses. Using the IMF’s prediction for global economic growth and the historical correlation between Gross domestic product (GDP) and infant mortality in the poor world, it may be estimated that hundreds of thousands more children died in 2020 than would have occurred in a pre-pandemic realistic situation [29]. In a single year, this would essentially undo the reduction in infant mortality that has occurred over the previous 2 to 3 years [46].

##### ***Safety***

For most youngsters, home symbolizes a source of protection and safety. The unfortunate situation is the reverse for a minority, however. Violence by caretakers is the most frequent kind of violence that children face. Such violent actions are more likely to happen when families are cooped up at home and under a lot of stress. Sixty percent of all children globally reside in nations that are either completely or partially under lockdown [31]. Tragically, lockdowns also provide child abusers the chance to hurt children. Rarely are children in a position to disclose such serious acts. Though social work and other relevant legal and protective services for children are being stopped or reduced, children during COVID had no longer access to teachers to report issues at home at a time when there is a greater need [47, 48].

##### ***Social isolation and sensory deprivation***

According to the analysis, social isolation is strongly linked to anxiety and depression in kids and teenagers. Cortisol levels rise and cognitive development is negatively impacted by social isolation. As a result, it is really concerning to see how children are developing cognitively, physically, and mentally throughout the epidemic. Isolation has been linked to a rise in memory loss in the elderly [49]. Early research on persons who are not exposed to sensory stimuli (visual, auditory, and tactual) reveals that over a short period of time, their cognitive abilities deteriorate and some start to experience hallucinations [50]. Hugs and handshakes help people feel more connected. Isolation and separation, which are used to stop the transmission of the coronavirus, increase susceptibility regardless of the source or aim [29, 51].

## How to prevent children from COVID-19

**Fig. 5:**
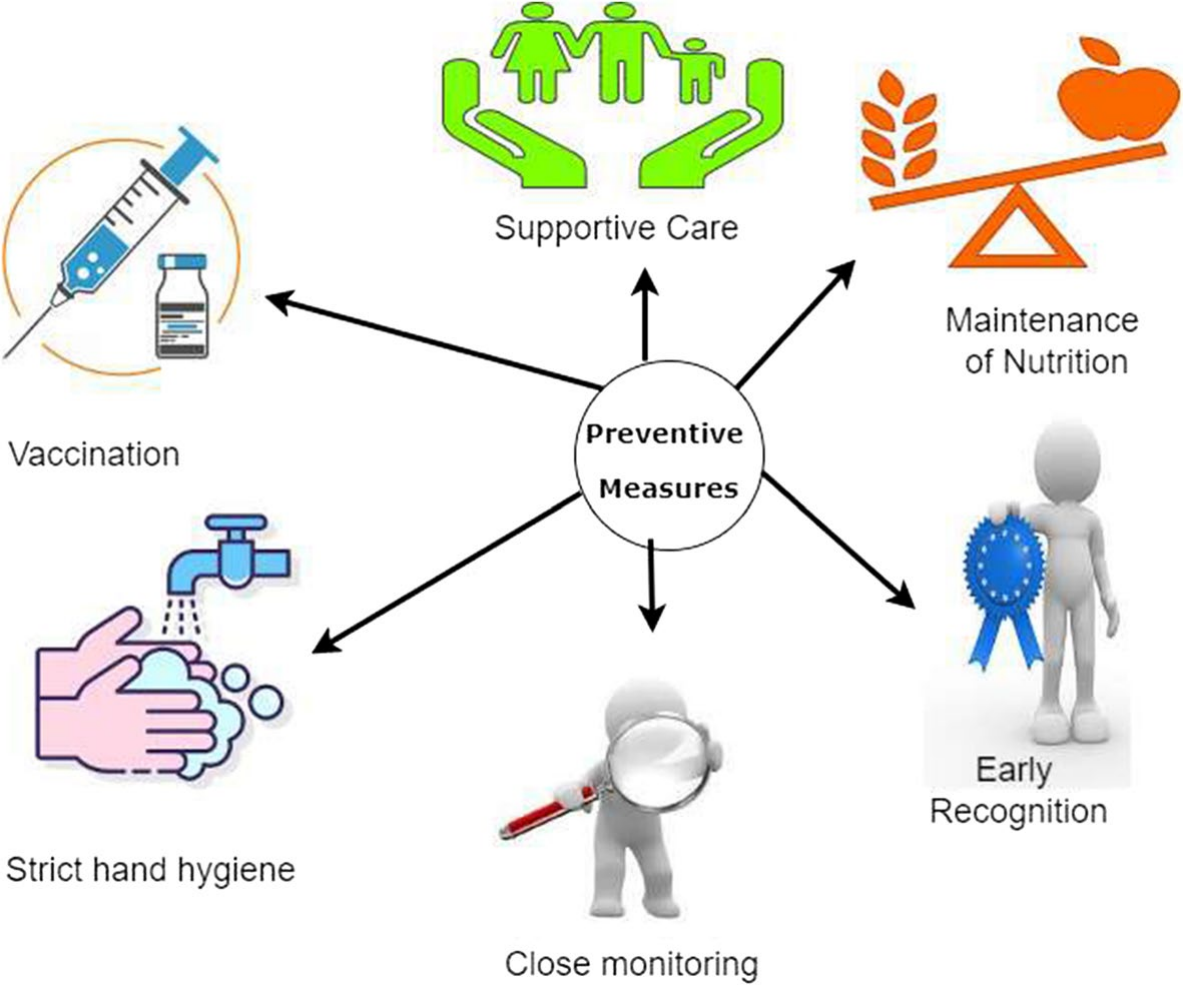
Preventive measures, the image was drawn by the author

Getting infected with COVID-19 does more harm than good. Given the clinical signs and problems, as well as the long-term mental and physical effects, it is preferable to avoid developing the condition in the first place [29]. It will assist us in avoiding the terrible stress brought on by the COVID-19 pandemic, as well as the other consequences brought on by COVID-19. Preventive measures are a group of publicly available strategies that can help to slow the spread of new coronaviruses [52, 53]. Wearing the proper mask, maintaining sufficient physical distance when in a crowd, avoiding touching public places, and not going out in public unless essential are some of the steps that can be successful in preventing the spread of the virus (Fig. 5). Some others are as follows:

### Early recognition

Gain an understanding of coronavirus illness, including manifestations, comorbidities, transmission, prevention, and management. Keep up to date on COVID-19 using reliable sources like United Nations International Children’s Emergency Fund, World Health Organization, and national health authority recommendations. Be cautious of misleading info that may be spread by viral marketing or the Internet [12].

### Close monitoring

Call your local health center for guidance and then bring your child in if necessary. Remember that COVID-19 symptoms like cough and fever can be confused with the flu or the common cold, which are far more frequent. Keep your ill child home and inform the school authority of his or her symptoms and absenteeism, so that learners are able to continue e-learning at home, seek reading, and homework. Explain what is going on to your child in simple terms and assure children that they are protected [22, 52].

### Supportive care

Stress can affect children in a variety of ways. Sleeping problems, enuresis, abdominal or headache, and being worried, distant, aggressive, touchy, or terrified of being alone are all common responses. Child’s reactions should be met with support, and they should be told that they are typical reactions to an unusual event. Pay heed to their fears and take the time to soothe and affectionate them, as well as reassure them that they are safe and constantly praise them. Create opportunities for children to play and relax if at all practicable. Maintain as many normal activities and routines as appropriate, especially before going to bed, or assist in the creation of new routines in new surroundings [8, 26].

### Vaccination

Vaccine for COVID-19 is an important preventative step in the fight against the pandemic. COVID-19 vaccinations are now readily accessible everywhere, and the CDC advises that everyone aged 12 and above get vaccinated. The US Food and Drug Administration (FDA) licensed an mRNA vaccine (Pfizer-BioNTech) as a 2-dose regimen for clinical COVID-19 prophylaxis in people aged 16 and above on August 23, 2021 [54]. This vaccine is also approved for use in children aged 12 to 15 years old as part of an Emergency Use Authorization (EUA). EUA has approved the use of a second mRNA vaccine (Moderna), as well as a recombinant, replication-incompetent adenovirus serotype 26 (Ad26) vector vaccine (Janssen vaccine [Johnson & Johnson]) [55].

### Strict hand hygiene

Hands should be washed often with soap and water. Use an alcohol-based hand sanitizer with at least 60% alcohol if soap and water are not readily available. If your hands are noticeably unclean, wash them with soap and water. Ensure that safe drinking water is provided, as well as clean and accessible toilets or latrines, at home. Ascertain that garbage is collected, stored, and disposed of safely. Avoid touching your face, eyes, mouth, or nose by coughing and sneezing into a tissue or your elbow [55, 56, 51].

### Maintenance of nutrition

A nutritious diet is especially crucial for children up to the age of 2 years to safeguard their immunity and promote their continued development. Because of the COVID-19 problem, parents and caregivers may be unable to afford or obtain the food that they regularly provide their young children. This could imply changing habits [26].

Breastmilk fulfills all of a child’s dietary requirements and protects them from disease until they are 6 months old. Additional drinks or meals are unnecessary for babies and may even be hazardous. Vegetables and fruit, grains, legumes, nuts, animal and dairy products, and “supply like rice must all be consumed by children starting at the age of 6 months. To keep hydrated, they must consume ample liquids such as breastmilk and clean water. Add one or two healthy snacks in between meals, as well as plenty of clean water, to keep kids going throughout the day. Snacks such as fruit and veggies that are soft or chopped into bite-sized pieces are ideal.

Formula feeds and commercial infant foods should be avoided. Families are more likely to stay at home because of the COVID-19 problem, which provides an opportunity to replace formula feed and manufactured meals with home-prepared foods. Young children can eat home-cooked food as long as it is nutritious, diverse, and prepared safely. It helps children learn to appreciate various flavors while saving money and promoting healthy living [57, 26].

## Conclusions

The pediatric age group is equally prone to the COVID-19 infection, but they show lesser manifestations of the disease and often recover rapidly, according to lessons learned from previous similar outbreaks of SARS and MERS and research completed to date. Critical care infrastructure, such as ICUs and oxygen support, as well as mechanical ventilation, are not required. However, with comorbid and congenital illnesses children are just as vulnerable as adults because they are already in an immunological state. They are required to be handled with caution. Almost all investigations have found that vertical transmission of the infection is unlikely, although additional research is needed. The psychological and behavioral aspects of children’s lives are frequently disregarded, but it is past time to address them as well. The absence of research on such matters is a big roadblock, and more and more studies examining the mental health of children in various settings must be conducted on a regular basis. Vaccination has proven to be an effective strategy against COVID-19, and it should be expanded to include children of all ages, as mutations in the novel coronavirus can damage anyone.

## Data Availability

Not applicable.

## Abbreviations

SARS: Severe acute respiratory syndrome
MERS: Middle Eastern respiratory syndrome
RT-PCR: Reverse transcriptase polymerase chain reaction
ICU: Intensive care unit
FDA: Food and Drug Administration
EUA: Emergency Use Authorization
PPP: Purchasing Power Parity
